# Dynamic Changes of Gut Microbial Communities of Bumble Bee Queens through Important Life Stages

**DOI:** 10.1128/mSystems.00631-19

**Published:** 2019-12-10

**Authors:** Liuhao Wang, Jie Wu, Kai Li, Ben M. Sadd, Yulong Guo, Daohua Zhuang, Zhengyi Zhang, Yanping Chen, Jay D. Evans, Jun Guo, Zhigang Zhang, Jilian Li

**Affiliations:** a Key Laboratory of Pollinating Insect Biology of the Ministry of Agriculture, Institute of Apicultural Research, Chinese Academy of Agricultural Science, Beijing, China; b College of Resources and Environmental Sciences, Henan Institute of Science and Technology, Xinxiang, Henan, China; c State Key Laboratory of Genetic Resources and Evolution, Laboratory of Evolutionary & Functional Genomics, Kunming Institute of Zoology, Chinese Academy of Sciences, Kunming, Yunnan, China; d State Key Laboratory for Conservation and Utilization of Bio-Resources in Yunnan, Yunnan University, Kunming, Yunnan, China; e School of Biological Sciences, Illinois State University, Normal, Illinois, USA; f Shenzhen Digital Life Institute, Shenzhen, China; g United States Department of Agriculture (USDA)—Agricultural Research Service (ARS) Bee Research Laboratory, Beltsville, Maryland, USA; h Faculty of Life Science and Technology, Kunming University of Science and Technology, Kunming, Yunnan, China; Pacific Northwest National Laboratory

**Keywords:** bumble bees, 16S rRNA locus, queens, physiological states, gut microbiome

## Abstract

Bumble bees are important pollinators in natural and agricultural ecosystems. Their social colonies are founded by individual queens, which, as the predominant reproductive females of colonies, contribute to colony function through worker production and fitness through male and new queen production. Therefore, queen health is paramount, but even though there has been an increasing emphasis on the role of gut microbiota for animal health, there is limited information on the gut microbial dynamics of bumble bee queens. Employing 16S rRNA amplicon sequencing and quantitative PCR, we investigate how the adult life stage and physiological state influence a queen’s gut bacterial community diversity and composition in unmated, mated, and ovipositing queens of Bombus lantschouensis. We found significant shifts in total gut microbe abundance and microbiota composition across queen states. There are specific compositional signatures associated with different stages, with unmated and ovipositing queens showing the greatest similarity in composition and mated queens being distinct. The bacterial genera *Gilliamella*, *Snodgrassella*, and *Lactobacillus* were relatively dominant in unmated and ovipositing queens, with *Bifidobacterium* dominant in ovipositing queens only. *Bacillus*, *Lactococcus*, and *Pseudomonas* increased following queen mating. Intriguingly, however, further analysis of unmated queens matching the mated queens in age showed that changes are independent of the act of mating. Our study is the first to explore the gut microbiome of bumble bee queens across key life stages from adult eclosion to egg laying and provides useful information for future studies of the function of gut bacteria in queen development and colony performance.

**IMPORTANCE** Bumble bee queens undergo a number of biological changes as they transition through adult emergence, mating, overwintering, foraging, and colony initiation including egg laying. Therefore, they represent an important system to understand the link between physiological, behavioral, and environmental changes and host-associated microbiota. It is plausible that the bumble bee queen gut bacteria play a role in shaping the ability of the queen to survive environmental extremes and reproduce, due to long-established coevolutionary relationships between the host and microbiome members.

## Abstract (precis)

Bumble bee queens undergo a number of biological changes as they transition through adult emergence, mating, overwintering, foraging, and colony initiation including egg laying. Therefore, they represent an important system to understand the link between physiological, behavioral, and environmental changes and host-associated microbiota. It is plausible that the bumble bee queen gut bacteria play a role in shaping the ability of the queen to survive environmental extremes and reproduce, due to long-established coevolutionary relationships between the host and microbiome members.

## INTRODUCTION

Our results show that there is a significant difference in diversity and composition of the gut microbial communities in queens of Bombus lantschouensis across different states. This study provides insight into the relationship between the bacterial community and the physiological states of bumble bee queens and lays the foundation for further studies of the functioning of the gut microbiota in the health and reproductive success of bumble bee queens.

Symbiotic bacteria play important roles in physiology, behavior, and ultimately fitness of their animal hosts, including insects (1, 2). They can supply the necessary nutrition for their host (3, 4), improve the host’s development and fecundity (5, 6), modulate their metabolism (7), and induce insects’ aggregations (8, 9) as well as determine kin recognition and mate choice (10–12). Conversely, host physiology and developmental stage can influence the composition of host-associated microbiota (13, 14). Bumble bees are social insects with annual colony cycles, and queens undergo a number of physiological and developmental changes as they progress through mating and diapause to subsequent oviposition and colony production (15, 16). However, there is limited information on the association between these changes and the critical gut microbiota of bumble bees.

Compared with the gut microbiota of many other animals, adult workers of social apid bees (bumble bees and honey bees) harbor a relatively simple yet specialized gut microbiota dominated by several recently described bacterial species, including Gilliamella apicola, Snodgrassella alvi, and specialized species of *Lactobacillus* (17–22). A number of beneficial interactions among these microbes and the honey and bumble bees have been reported, including increased metabolic functionality, protection from invading pathogens through facilitation of the immune response (23, 24), or exclusionary effects (25–27).

In contrast to worker bees, few studies have examined microbial communities that are associated with honey bee and bumble bee queens, even though their health and proper function are central to the productivity of their colonies. Parmentier et al. (28) found that typical core gut microbial communities in adults are absent in the larvae of wild bumble bees, which suggested that the compositions of microbial communities are different among different developmental stages or castes of bumble bees. The microbiota has also been shown to change during the developmental trajectory of honey bee queens (29) and the hibernation of bumble bee queens (30). Tarpy et al. (29) suggested that mainly enteric bacteria are present in honey bee queens at an early stage, with the predominant gut bacteria being *Alphaproteobacteria* at maturity, and yet the size and composition of workers’ symbiotic bacteria are relatively stable across ages. Likewise, through an isolated queen experiment, Powell et al. (31) found fewer of the primary honey bee microbiota in honey bee queen guts, and the species of bacteria were different from those of workers. Queens of honey bees and bumble bees have distinct biology. The latter goes through diapause and has a solitary founding stage, neither of which occurs in honey bees. These differences and the fact that founding bumble bee queens are the source of certain components of the gut microbiota of workers in the subsequent colony (32) make understanding the dynamics of the gut microbiota across key life stages of bumble bee queens crucial. In bumble bee queens, Bosmans et al. (30) revealed that the bacterial community composition during hibernation is richer, including some psychrophilic and psychrotrophic taxa, than in nonhibernating active queens. These studies indicate that changes of gut microbiota of honey bee and bumble bee queens may be associated with the physiological variation and developmental stage. However, temporal dynamics of bumble bee queen gut microbiota remain underexplored during sexual maturity, and patterns may provide novel insights into the interplay between queen development and physiology and the queen’s microbiota, the change to which may offer feedback on microbiota functioning in queen hosts.

Bumble bees are important pollinators of many flowering plant species in temperate to subarctic and alpine zones (33). In recent decades, many bumble bee species have been identified as declining, particularly in Europe and North America (34). Many factors have been suggested to be responsible for these declines (35). With ongoing land use and climate changes, some bumble bee species have also been predicted to become critically endangered and vulnerable in central mainland China and northeastern Asia in the future (36). At the same time, techniques for artificial hibernation and large-scale propagation have been developed that enable the commercial production of bumble bee colonies in the hundreds of thousands annually (37). Population declines and the agricultural importance of bumble bees necessitate a greater understanding of factors implicated in bumble bee health, such as the gut microbiota and their health-related functions.

**FIG 1:**
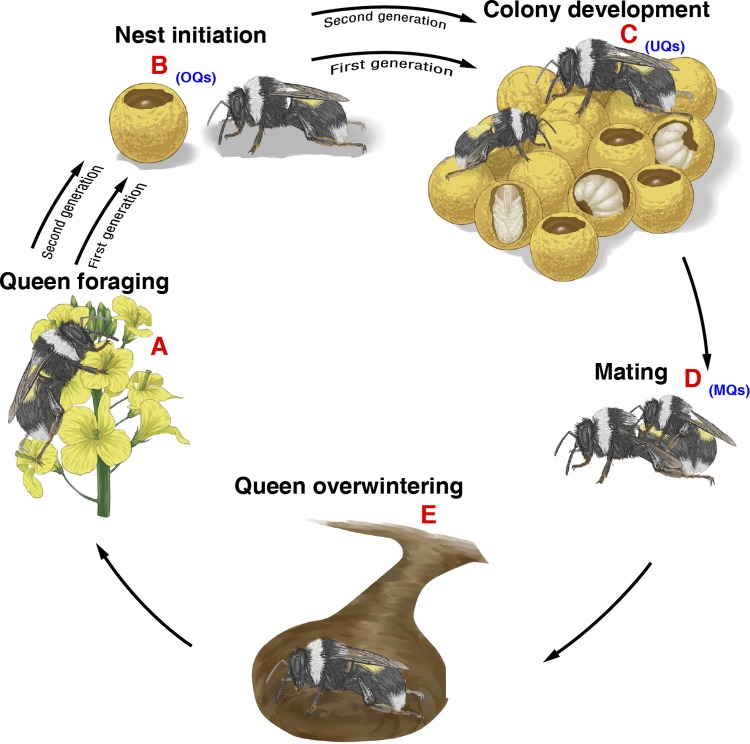
Bumble bee life cycle. In the wild, *Bombus lantschouensis* queens emerge from hibernation in spring, forage (A), and find a nesting location in which to lay eggs and initiate a new colony (B). For the initiation of colonies to produce experimental queens in the laboratory, spring queens were collected from the field (first generation). The worker population grows, and toward the end of the colony cycle in late summer, sexuals (virgin queens and males) are produced (C). Young queens mate with only one male (D) and subsequently hibernate to produce the next generation (E, second generation B). In this study, we assessed three stages of *Bombus lantschouensis* queens: unmated queens (UQs; virgin queens) following queen eclosion to adulthood in colonies (C), mated queens (MQs; mating successfully with drone, D), and ovipositing queens (OQs; queens actively laying eggs, second generation B).

Temperate bumble bees have an annual eusocial life cycle, with a solitary queen phase between mating and the foundation of new colonies in spring following the emergence of queens from hibernation (16). Toward the end of the colony cycle in late summer, sexuals (virgin queens and males) are produced. Young queens mate, usually with a single male for many species; hibernate; and subsequently emerge to produce the next generation (Fig. 1) (38, 39). The queen is critical to the development of the microbiota of individuals within the colony, with vertical transmission of certain core gut microbes occurring from mother to offspring (32). The composition of the queen’s microbiota and its contribution to health are also critically important given that the queen is the principal reproductive female in a colony, playing a crucial role in colony development and longevity (40–42).

A better understanding of the composition of gut microbiota in different physiological states of bumble bee queens would shed light on the complex interplay between the microbiota and queen health. To this end, using an amplicon sequencing approach, we assessed microbiota composition in three queen types of the bumble bee *Bombus lantschouensis*: unmated queens (UQs; virgin queens) 7 days after adult eclosion; mated queens (MQs), mated at 7 days posteclosion and sampled 7 days later; and posthibernation ovipositing queens (OQs; queens actively laying eggs). These queens were offspring from laboratory-reared colonies (Fig. 1) and fed a controlled diet. To confirm differences, targeted quantification PCR (qPCR) was carried out on dominant bacterial genera identified in the three groups. This analysis included a temporal analysis of abundances comparing unmated (1 to 7 days posteclosion) and mated (1 to 7 days postmating) queens. To further distinguish intrinsic changes from those associated with mating itself, abundances were also assessed in another group of unmated queens sampled every 2 days between 1 and 15 days posteclosion. Our study is the first to explore dynamic changes of the gut microbiota across important life stages of bumble bee queens, from adult eclosion to colony foundation and egg laying. It shows dynamic diversity and variation of gut bacterial communities and improves our understanding of possible relationships between the gut microbial communities and different developmental and physiological states of bumble bee queens.

## RESULTS

### 16S rRNA gene sequencing analysis and taxa generated.

We processed and filtered sequences, clustered them into operational taxonomic units (OTUs) with 97% minimum identity, and excluded plastids, singletons, and OTUs restricted to single samples. A total of 2,107,642 sequences were obtained in 86 samples including unmated queens (UQs; *n* = 30), mated queens (MQs; *n* = 27), and ovipositing queens (OQs; *n* = 29), which range from 9,915 to 44,451 (24,507 ± 961, mean ± standard error [SE]) per sample. These sequences were clustered into 390 OTUs, with a range of 17 to 138 per sample (67 ± 4, mean ± SE). The core OTUs comprised approximately 17.94% of the total candidates, while 282, 186, and 175 OTUs were identified uniquely in the UQ, MQ, and OQ groups, respectively (see Fig. S1 in the supplemental material).

10.1128/mSystems.00631-19.1 FIG S1 Common and specific OTU distribution of gut microbiota among three groups of queens. Download FIG S1, PDF file, 0.05 MB. Copyright © 2019 Wang et al. 2019 Wang et al. This content is distributed under the terms of the Creative Commons Attribution 4.0 International license.

To better understand the differences in the microbiome between the three queen stages, OTU sequences were blasted against the annotated SILVA 16S rRNA reference database (https://www.arb-silva.de). Twenty phyla were detected across all samples. However, four bacterial phyla accounted for more than 99% of all sequences. Ranked by relative abundance, these phyla were *Proteobacteria* (66%), *Firmicutes* (26%), *Actinobacteria* (6%), and *Bacteroidetes* (1%). Unclassified bacteria at the phylum level were rare and represented less than 1% of all sequences (Fig. S2).

10.1128/mSystems.00631-19.2 FIG S2 Relative abundance of predominant gut bacterial phyla in three groups of queens. Download FIG S2, PDF file, 0.03 MB. Copyright © 2019 Wang et al. 2019 Wang et al. This content is distributed under the terms of the Creative Commons Attribution 4.0 International license.

### Estimates and variations of microbial local diversity among samples from three stages of queens.

**FIG 2:**
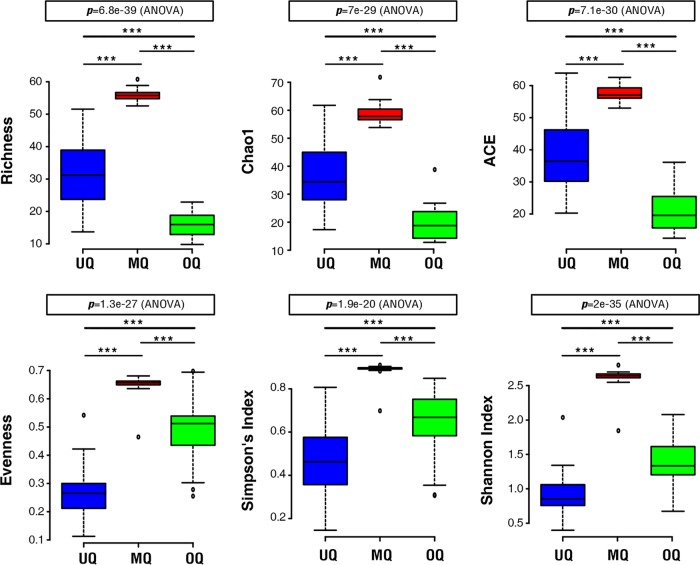
The microbial community diversity of different queen states. Box plots show OTU measures of raw richness, ACE, Chao1, evenness, and Simpson and Shannon diversity indices. *** indicates significant differences among groups (*P* < 0.0001). UQ, unmated queen; MQ, mated queen; OQ, ovipositing queen.

We employed three species-richness measures of richness, Chao1, and abundance-based coverage estimator (ACE) to investigate the number of different OTUs (i.e., species richness) between queen groups. All measures gave qualitatively similar results, with the richness significantly affected by group identity (Fig. 2). Gut microbial communities from the MQ state had the highest richness, followed by UQs, and OQs with the lowest (*P* < 0.0001). Evenness was also calculated for the microbial communities, to investigate how the queen status influenced the equality of distribution of different microbes within each gut. The highest species evenness was observed for the MQ state, indicating that abundances of the diverse gut microbes associated with the MQ state were the most evenly distributed (Fig. 2). Unlike richness, the evenness of communities of OQs was greater than those of UQs. Finally, Simpson and Shannon diversity indices were calculated incorporating richness and evenness. The results for overall diversity mirrored those for evenness, with MQs having the greatest diversity, followed by OQs, and UQs having the lowest (Fig. 2). The evenness and diversity results indicate that the gut microbial community of the unmated queen is dominated by only a few species. Indeed, the bacterial genera *Gilliamella* and *Snodgrassella* were the two most dominant gut microbiota taxa, accounting for 82.5% ± 3.49% (mean ± SE) of all total sequence reads in unmated queens. In brief, our results suggest variation in the gut microbial community structure of the three queen stages (UQ, MQ, and OQ).

**FIG 3:**
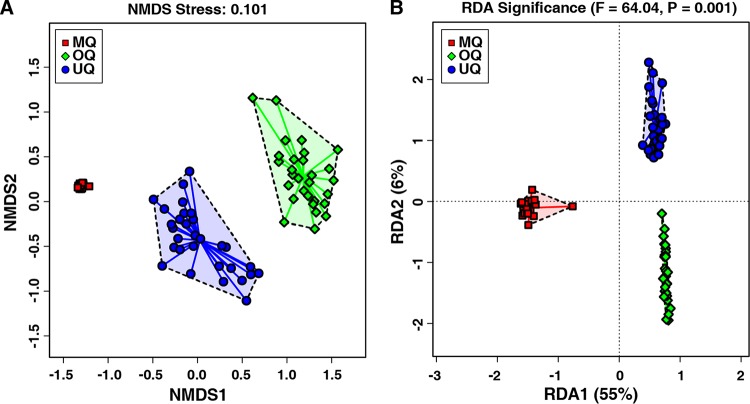
The similarity and variation of gut microbial community structures across the three queen groups of unmated queens (UQ), mated queens (MQ), and ovipositing queens (OQ). (A) Unsupervised NMDS plot of beta-diversity (Bray-Curtis dissimilarity) showing clustering of the gut microbiota from individual samples (*n* = 86). Distances between individual samples reflect the extent of the similarity of gut microbiota. (B) Supervised RDA of the relationship between queen states and the relative abundance of bacterial genera. The *P* value above the figure indicates that the variable (UQ, MQ, and OQ) significantly explains variation in sample distances.

To test the above inference, we conducted beta-diversity analysis of the microbial communities among UQ, MQ, and OQ states using both unsupervised and supervised methods. The unsupervised nonmetric multidimensional scaling (NMDS) plots (Bray-Curtis distance matrix) revealed a clear separation of samples according to the different bumble bee queen stages (stress = 0.101 provides a good representation in reduced dimensions) (Fig. 3A). The supervised redundancy analysis (RDA) further indicated that as the queens progress through the different physiological states, these can significantly influence the gut microbial community composition of the host (*P* = 0.001, Fig. 3B). These results obtained by two independent methods to assess beta-diversity consistently suggested that there were significant structural separations of the gut microbiota among UQs, MQs, and OQs. The microbial community structure associated with the MQ state was significantly distinguished from UQ and OQ states (implicated by RDA1 with 55% of variation in Fig. 3B). Enterotype analysis of the genus-level table for the microbial communities of all samples also resulted in an optimal number of enterotypes (clusters of similar communities) of three (Fig. S3). This finding indicates that the gut microbial community structure of mated queens is unique and may be a consequence of physiological changes associated with mating itself or the development of the microbiota as the queen ages or moves toward diapause. However, the greater similarity between UQs and OQs suggests that the shift is more likely to be associated with changes during the time or process of mating.

10.1128/mSystems.00631-19.3 FIG S3 Principal-component analysis (PCA) plot of enterotype clusters observed in *Bombus lantschouensis* on three groups of queens (*n* = 86). Download FIG S3, PDF file, 0.4 MB. Copyright © 2019 Wang et al. 2019 Wang et al. This content is distributed under the terms of the Creative Commons Attribution 4.0 International license.

### The discovery of gut microbial biomarkers associated with different queen states.

**FIG 4:**
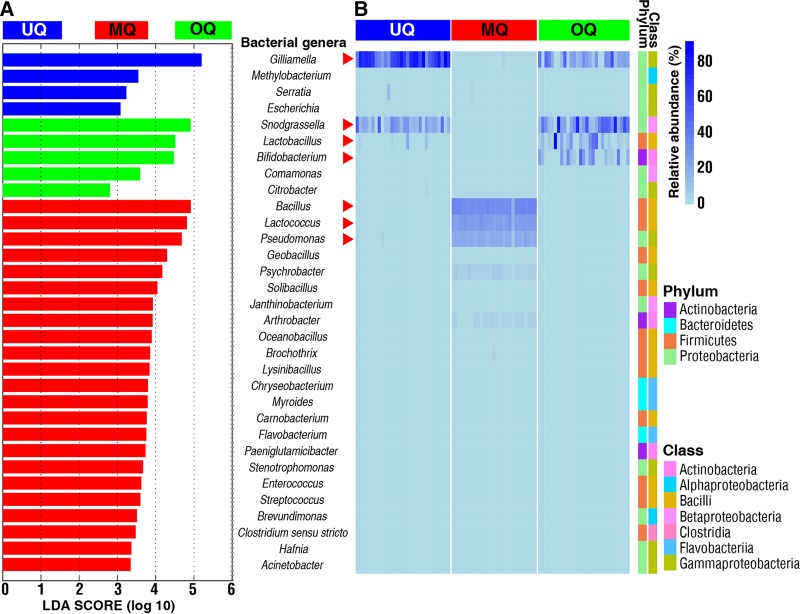
Comparison of the predominant bacterial genera among the three queen groupings of unmated queens (UQ), mated queens (MQ), and ovipositing queens (OQ). (A) LEfSe analysis indicates significantly different abundances of bacterial genera for each group. LDA score value is 2. (B) Heatmap depicts the relative abundances of the identified gut bacterial genera across the different queen groupings. Relative abundance of the gut bacterial genera *Gilliamella*, *Snodgrassella*, *Lactobacillus*, *Bifidobacterium*, *Bacillus*, *Pseudomonas*, and *Lactococcus* among three groups with significant differences (*P* < 0.05, Kruskal-Wallis tests). qPCR analyses were used to validate relative abundances of the seven differentially abundant genera marked by red arrowheads.

Specific gut microbiota features can reflect specific disease or normal physiological conditions of the host (43). Hence, we analyzed the relative abundance of four phyla and their distribution in the guts of three stages of queens (Fig. S2). *Proteobacteria* ware the most predominant gut bacterial phylum in both UQs (96.4% ± 0.77%, mean ± SE) and OQs (72.2% ± 4.42%, mean ± SE). In contrast, *Firmicutes* account for 65.5% ± 1.16% (mean ± SE) of total reads in MQs. These results indicate potential broad-scale gut microbial markers unique to the three queen stages. Using LEfSe analysis, we identified gut microbiota features specific to the three queen groups (Fig. 4A). Among them, there were four bacterial genera in UQs, five genera in OQs, and 23 genera in MQs (Fig. 4A). Combined with a heatmap view of the relative abundance of gut bacterial genera, our results show that seven bacterial genera can be used as signatures of the three queen states (Fig. 4B). These genera showed significant differences in relative abundance between queen states (*P* < 0.05, Kruskal-Wallis test). *Gilliamella* and *Snodgrassella* were abundant bacterial genera associated with both UQs and OQs, with high abundances of *Lactobacillus* and *Bifidobacterium* also associated with OQs. The main bacterial genera in mated queen (MQ) guts were *Bacillus*, *Lactococcus*, and *Pseudomonas*. Besides the identified highly abundant bacterial phylotypes for the MQ state, there were also low-abundance bacterial genera associated with MQs, including *Proteobacteria* (*Psychrobacter*, *Methylobacterium*, *Serratia*, *Escherichia*, *Comamonas*, *Citrobacter*, *Janthinobacterium*, *Stenotrophomonas*, *Brevundimonas*, *Hafnia*, and Acinetobacter), *Firmicutes* (*Brochothrix*, *Oceanobacillus*, *Geobacillus*, *Solibacillus*, *Lysinibacillus*, *Carnobacterium*, *Enterococcus*, *Streptococcus*, and *Clostridium sensu stricto*), *Actinobacteria* (*Arthrobacter* and *Paeniglutamicibacter*), and *Bacteroidetes* (*Myroides*, *Chryseobacterium*, and *Flavobacterium*) (Fig. 4). The relatively diverse compositions in MQs were consistent with the finding of the highest alpha-diversity being in MQs, as presented in Fig. 2. These results demonstrate specific gut microbial features associated with queens at the postmating stage.

### Copy number validation of differentially abundant bacterial genera in the three queen stages.

**FIG 5:**
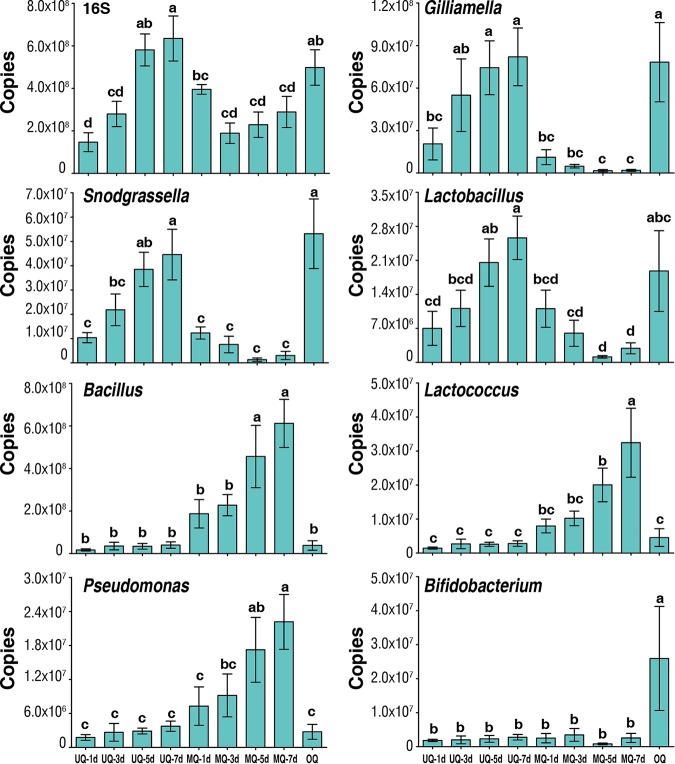
The change of copy numbers of overall bacteria (16S rRNA copies) and bacterial genera at different time points of the three queen groupings. UQ, unmated queen; MQ, mated queens; OQ, ovipositing queens. Days (d) for UQ represent days posteclosion and for MQ represent days postmating, with mating occurring at 7 days posteclosion. Bars represent means ± SEM. Different letters above bars within plots represent significant differences (pairwise *t* tests, *P < *0.05).

**FIG 6:**
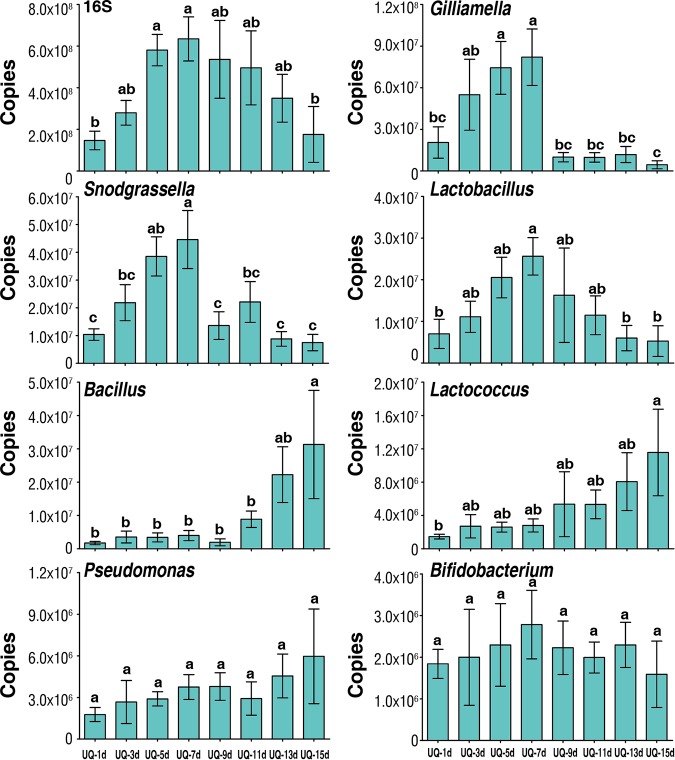
The change of copy numbers of overall bacteria (16S rRNA copies) and bacterial genera at different time points for UQs (unmated queens). Days (d) represent days posteclosion. Bars represent means ± SEM. Different letters above bars within plots represent significant differences (pairwise *t* tests, *P < *0.05).

To verify the accuracy of culture-independent analysis of bacterial genera described by Fig. 4, we used 16S rRNA gene-targeted group-specific primers for real-time PCR analysis of seven identified predominant bacterial genera (*Gilliamella*, *Snodgrassella*, *Lactobacillus*, *Bifidobacterium*, *Bacillus*, *Lactococcus*, and *Pseudomonas*) in unmated queens, mated queens, and ovipositing queens. The mean absolute number (± SD) of the overall bacterial rRNA genes of each queen stage and age was estimated to vary from 1.47 × 10^8^ (± [4.5 × 10^7^]) to 6.35 × 10^8^ (± [1.1 × 10^8^]) copies per gut, with each of the predominant bacterial genera differentially contributing to this total (Fig. 5 and 6).

This targeted approach confirmed the patterns seen in Fig. 4 for UQs 7 days posteclosion, MQs 7 days after mating, and OQs (Fig. 5). In addition, we investigated the temporal dynamics of changes within UQ, OQ, and MQ groups. *Gilliamella*, *Snodgrassella*, and *Lactobacillus* increased with age posteclosion (1 to 7 days) in UQs and declined significantly following mating at 7 days across the MQ stage (1 to 7 days postmating) but rebounded to the peak seen in UQs 7 days posteclosion by the time of queen egg laying (OQ). This initial increase, decline, and rebound were also present in the total 16S rRNA copies (Fig. 5), indicating changes in total bacterial abundance. However, the decline from 7 days posteclosion was not as pronounced due to increases in other genera. *Bacillus*, *Lactococcus*, and *Pseudomonas* increased postmating from low levels in the UQ state, returning to UQ state abundances in OQs. Uniquely, the bacterial genus *Bifidobacterium* was found at higher abundances only in OQs.

To further understand the relationship of microbiota composition with queen state and age, the same bacterial genera and total bacterial abundance were quantified in unmated queens at eight time points from 1 to 15 days posteclosion (Fig. 6). Interestingly, the results for all genera qualitatively mirrored those across UQ and MQ states shown in Fig. 5, even though these queens remained unmated throughout the time period. This indicates that the changes in microbiota composition in mated queens are not entirely the result of mating *per se* but rather are the consequence of age- or physiology-related changes as queens reach maturity and approach hibernation.

## DISCUSSION

Our study demonstrates temporal dynamics in the gut microbiota of bumble bee queens at 1 to 15 days post-adult eclosion and posthibernation, comparing three physiological states: unmated, mated, and ovipositing. Over the first week following eclosion, we see increases in three well-known apid bee symbionts, *Gilliamella*, *Snodgrassella*, and *Lactobacillus*, which have previously been reported in worker bumble bees (44) and also show such a similar temporal increase in honey bee workers (45). The prehibernation peak of these three bacterial genera is consistent with sexual maturity in bumble bee queens, indicating their potential to functionally contribute to the promotion of physiological development of virgin bumble bee queens. Surprisingly, independent of mating status, after the 7-day peak, these early-abundant three bacterial genera were dramatically replaced by other bacteria, such as those members belonging to the *Bacillus* genus. The independence of this change from actual mating suggests that the shift may be the consequence of age- or physiology-related changes as queens reach maturity and approach hibernation. This change is particularly interesting given that the predominance of *Gilliamella*, *Snodgrassella*, and *Lactobacillus* was restored in posthibernation ovipositing queens. Additionally, an increase in *Bifidobacterium* is unique to the ovipositing queen group. *Bifidobacterium* increases during late pregnancy in humans, with a potential beneficial role (46, 47). Overall, the dynamic nature of the microbiota of bumble bee queens, including of some core and functionally important taxa, suggests links to the specific biology of bumble bee queens.

Whether gut microbiotas influence sexual maturation of their animal hosts remains little explored. Our results imply a connection between a change in the microbiota of bumble bee queens and the period of sexual maturity, but causation cannot be inferred. It has been shown that the mouse microbiota is required for sex-specific diurnal rhythms of gene expression and metabolism, showing that it plays a key role in ensuring proper sexual maturation and growth hormone secretion (48). In addition, studies have found that gut symbionts have potential effects on reproductive behaviors in insects. For example, in Drosophila melanogaster commensal bacteria play a role in mating preferences (10) and alteration of female microbiota counteracts a default male outbreeding strategy by inhibiting female sexual signaling (49). The gut microbiota has also been shown to modify olfactory sense-guided microbial preferences and foraging decisions in *Drosophila*, indicating a role of animal microbiota in shaping host fitness-related behavior through their chemosensory responses (50). Moreover, the gut bacterium Lactobacillus brevis has been found to modulate locomotor activity in D. melanogaster, which is mediated by the level of a sugar and the activity of neurons that produce the molecule octopamine (51). While the changes in the microbiota around the time of mating that we have uncovered in our study are intriguing, further work would be required to elucidate if the microbiota influences bumble bee queen mating behavior and chemical communication required for copulation, such as queen sex pheromone production (52).

Disruption of the gut microbiota of primary termite reproductives has been shown to have negative consequences for reproduction (53). A similar connection has been made between microbiota presence and parthenogenetic reproduction in *Daphnia* water fleas (54). Unlike these experimentally induced disturbances of the microbiota, our results show a significant but naturally occurring change in the bumble bee queen microbiota. State-related changes have been shown for humans, with a shift in the gut microbiota during pregnancy (55–57), but our study is one of the first to report such a dramatic shift in an insect. These alterations could be adaptive, with positive effects on physiological development and behavior, or simply a side effect of intrinsic physiological changes ongoing within queens as they mature or of interactions with diet (58). Of importance for understanding the causes and consequences of these dynamic changes is the demonstration that while changes occurred around the time of mating, they are not the result of mating itself. However, further investigations are required to infer causation and any consequences of the decrease of earlier core bacteria and enrichment of *Bacillus*, *Pseudomonas*, and *Lactococcus* in the mature bumble bee queen. There is a potential for a direct active role of these enriched genera. Sabaté et al. (59) showed that Bacillus subtilis strains isolated from honey bee gut produced surfactins and fungicides that can inhibit the important honey bee pathogens Paenibacillus larvae and Ascosphaera apis. Also, *Bacillus* species can produce amylase that helps in the processing of flower nectar into honey in honey bees (60). In rotifers, *Lactococcus* was found to serve as a probiotic to enhance growth and immunity (61, 62). *Pseudomonas* species in insects have been shown to be involved in detoxification (63) and digestion through amylolytic, xylanolytic, and diazotrophic activities that could contribute to the nutritional supplement and nitrogen balance (64, 65).

A particularly intriguing finding is that the changes seen in the microbiota after 7 days posteclosion in both mated and unmated queens are reversed in ovipositing queens posthibernation. This restoration of the dominance of core gut microbes including *Gilliamella*, *Snodgrassella*, and *Lactobacillus*, in addition to an increase in *Bifidobacterium*, is likely critical for colony success, given the key role that many of these taxa play in bees. Hibernation constitutes a period of considerable environmental and physiological changes, yet relatively little is known about the relationship between gut microbiota and hibernation. However, hibernation has been shown to be associated with changes in the microbiota of some organisms (66–68). For example, Sommer et al. (68) showed that the microbiota and serum metabolites in brown bears differ seasonally between hibernating and active phases and that transplants of the specific microbiota into mice transferred some of the seasonal metabolic features seen in bears. For temperate bumble bees, hibernation is usual after mating and before oviposition as an adaptation to challenges imposed by winter. This period of diapause is associated with many changes in metabolism and physiology in general, alongside the environmental alterations (15). A difference in the microbiota between queens before and during hibernation has been observed (30), which could be associated with the period of hibernation itself. However, our observations indicate that significant changes to the gut microbial community of bumble bee queens occur prior to entrance into hibernation and are largely reset in queens following hibernation, when they are egg laying. Bumble bee queens utilize storage of energy, such as lipids and glycogen, to survive low winter temperatures (69). Similar to the work of Bosmans et al. (30), we detected some cold-loving and cold-tolerant bacterial genera, such as Acinetobacter, *Chryseobacterium*, *Hafnia*, *Psychrobacter*, and *Pseudomonas*, in samples of mated queens and also older unmated queens prior to hibernation. Their presence, even prior to the initiation of abiotic environmental changes associated with hibernation, could support the queens during the environmental transition during hibernation. The contribution of this distinct microbial community to hibernation success, relative to earlier and later microbial community compositions, is thus important to investigate further.

The microbiotas associated with organisms may be closely linked with physiological and behavioral changes in their hosts, either responding to these changes indirectly or directly being involved in them. Bumble bee queens undergo a number of biological changes as they transition through adult emergence, mating, overwintering, foraging, and colony initiation including egg laying. Therefore, they represent an important system to understand the link between physiological, behavioral, and environmental changes and host-associated microbiota. It is plausible that the bumble bee queen gut bacteria play a role in shaping the ability of the queen to survive environmental extremes and reproduce, due to long-established coevolutionary relationships between the host and microbiome members. Our results show that there is a significant difference in diversity and composition of the gut bacterial species between bumble bee queens at different ages and physiological stages. This provides a critical insight into the relationship between the bacterial community and queen status in bumble bees and establishes the basis for further work to determine if the microbiota changes identified are causal in the health and success of critically important bumble bee queens.

## MATERIALS AND METHODS

### Overview of sampling.

An approach using Illumina amplicon sequencing of the V3-V4 region of bacterial 16S rRNA was used to investigate differences in the microbiomes of queens at the different developmental stages of virgin unmated queens (UQs; 7 days post-adult eclosion, *n* = 30), mated queens (MQs; mated at 7 days posteclosion and sampled 7 days later, *n* = 27), and ovipositing queens after diapause (OQs; *n* = 29). A targeted approach focusing on bacterial genera to verify these results by qPCR and to additionally assess temporal changes was carried out in UQs at 1, 3, 5, and 7 days post-adult eclosion and in MQs at 1, 3, 5, and 7 days postmating, taking place 7 days posteclosion (*n* = 5 per time point). Furthermore, to investigate changes independent of mating, a final temporal assessment of UQs was carried out covering the period when MQs were sampled (1, 3, 5, 7, 9, 11, 13, and 15 days posteclosion, *n* = 5 per time point).

### Queen stages and sample collection.

Queens of *B. lantschouensis* were collected in the spring of 2016 from natural populations in Gansu and Ningxia provinces of China and identified by morphology and molecular methods (70, 71). Since the animals investigated in this study are neither vertebrates nor regulated invertebrates, ethical approval was not required, and the bees were sampled on property in Gansu and Ningxia provinces with consent of the manager of the Botanical Garden. Collected queens were reared in small plastic cages in the dark at a temperature of 27 ± 1°C and relative humidity of 50 to 60%. Sugar water (1:1, vol/vol) and apricot pollen were provided *ad libitum* to 100 colonies subsequently produced until males and gynes (new queens) emerged. Sampling for UQs, MQs, and OQs was carried out as outlined above. For matings, at 7 days post-queen eclosion, queens and males were kept at a ratio of 1:2, respectively, in a 4-m by 3-m by 2-m (length by width by height) net enclosure to ensure that one queen would mate with one male. For sampling of postdiapause OQs, mated queens were reared in a small wooden box until they became less active; they were then transferred to 4°C for diapause. After 4 months, they were revived and fed in the dark under the rearing conditions described above. Queens that had laid eggs and whose first batch of workers had emerged were collected for the OQ group. All collected samples were snap-frozen in liquid nitrogen and then stored at −80°C until the subsequent molecular analyses.

### Extraction of the gut DNA.

Before removing the whole gut from queens, including crop, midgut, ileum, and rectum, the sample surface was sterilized individually with 70% and 90% ethanol solution for 1 min, respectively, followed by multiple washes using double-distilled water. The abdomen was dissected with sterilized scissors and tweezers, and the whole alimentary canal was removed and transferred into a 1.5-ml microcentrifuge tube filled with 100 μl double-distilled water and ceramic beads (0.1 mm) for the subsequent DNA extraction.

Gut samples were homogenized in a tissue lyser (Qiagen, Hilden, Germany) followed by genomic DNA isolation using the Wizard Genomic DNA purification kit (Promega; A1120) according to the manufacturer’s instructions, with DNA suspended in 30 μl nuclease-free water. The concentration and quality of extracted DNA were assessed using a Qubit fluorometer (Invitrogen, Carlsbad, CA, USA) and 2% agarose gel electrophoresis, respectively. Extracted DNA was stored at −20°C until further processing.

### Illumina sequencing and bioinformatics analysis.

The hypervariable V3-V4 region of the bacterial 16S rRNA gene was amplified with the primers 341F (5′-CCTAYGGGRBGCASCAG-3′) and 806R (5′- GGACTACNNGGGTATCTAAT-3′) (72). Twenty-microliter PCR mixtures were set up with 4 μl 5× FastPfu buffer, 2 μl deoxynucleoside triphosphates (dNTPs) (2.5 mM), 0.8 μl each primer, 0.4 μl FastPfu polymerase, and template DNA (10 ng). Reactions proceeded in a GeneAmp 9700 (ABI) thermocycler with 95°C for 5 min; 27 cycles of denaturation at 95°C for 30 s, annealing at 55°C for 30 s, and elongation at 72°C for 45 s, followed by an additional elongation at 72°C for 10 min; and a dissociation stage at the end of the run. PCR products were detected by 2% agarose gel electrophoresis and purified using the QIAquick gel extraction kit (Qiagen). Library pools were constructed with equal amounts of each PCR product by using the TruSeq Nano DNA LT sample prep kit (Illumina), which was amplified through paired-end sequence on the Illumina MiSeq platform.

Raw Illumina sequence reads were modified by filtration, merging, and quality control, and barcode and primer sequences were removed, leaving library-specific tags. The fastq-join method was used to merge sequences using QIIME software (73), with an overlap length larger than 10 bp and mismatch ratio lower than 20%. Operational taxonomic unit (OTU) analysis was performed using the Uparse package (version 7.0.1001) with a 97% sequence identity on the basis of the effective tags (74). Each OTU was taxonomically assigned based on the SILVA 16S rRNA reference database using the assign_taxonomy.py program (http://qiime.org/scripts/assign_taxonomy.html). OTUs were processed by removing chloroplast sequences, mitochondrial sequences, and unclassified sequences and then obtaining species annotation information (confident threshold value, >0.8) (75, 76). Statistical differences in relative abundances of OTUs in different samples were analyzed by a nonparametric Kruskal-Wallis test, with analyses carried out using SPSS (version 17). The OTUs with relative abundance values of >0.001% (above three tags per sample) in at least one sample were retained.

The bacterial community diversities of gut samples were calculated and analyzed using the online software Calypso (http://cgenome.net/wiki/index.php/Calypso) with square root-based normalization of relative abundance. After samples were rarefied to even read depth, alpha-diversity measures of richness (Chao1 and ACE), evenness, and diversity indices (Simpson and Shannon) were compared between unmated, mated, and ovipositing queens by ANOVA. To determine if there are significant difference of gut microbial community structures among the three bumble bee queen states, the unsupervised nonmetric multidimensional scaling (NMDS) analysis of beta-diversity (Bray-Curtis distance matrix) was first conducted (77). The supervised redundancy analysis (RDA) was used to further validate complex associations between community composition and multiple explanatory variables (i.e., unmated, mated, and ovipositing queens in our study) (78). The *P* value reported indicates if each explanatory variable is significantly associated with variation in gut microbial composition. Gut bacterial genera associated with different physiological conditions of bumble bee queens were further identified using the linear discriminant analysis (LDA) effect size method (LEfSe) with default parameters (79). Enterotype analysis was carried out as in a previous study (44).

### Genus-specific primer design and PCR amplification.

16S rRNA gene sequences of key bacterial genera were retrieved from the GenBank database. The software DNAMAN was used to align and analyze sequences and identify highly conserved regions for designing primer pairs that were unique for each genus, using Primer Premier, version 5.0. Primer sequences of *Bacillus*, *Pseudomonas*, and *Lactococcus* were BacF (GATGCGTAGCCGACCTGAGA) and BacR (GGCGTTGCTCCGTCAGACTT), PseF (CCGTAACTGGTCTGAGAGGATG) and PseR (GCATGGCTGGATCAGGCTTT), and LactF (GCGATGATACATAGCCGACCTG) and LactR (AGTTAGCCGTCCCTTTCTGGTT), respectively. Primers for *Gilliamella*, *Snodgrassella*, *Lactobacillus*, and *Bifidobacterium* were from the previous study (80–82), as were the universal 16S rRNA primers to determine overall bacterial load in each queen gut sample (83, 84). To confirm the specificity of each bacterial primer set, 20-μl PCR amplification was performed in a reaction mixture containing 10 μl of SYBR Premix *Ex Taq* II (Tli RNase H Plus) (2×), 0.8 μl of the forward primer (10 μM), 0.8 μl of the reverse primer (10 μM), 1 μl of DNA sample, and 7.4 μl of double-distilled water. The PCR cycling conditions were as follows: predenaturation at 95°C for 30 s, followed by 40 cycles of denaturation at 95°C for 5 s and annealing at 60°C for 30 s, with a subsequent melt curve to check the specificity of the amplified fragments. The product sizes of PCR amplification were confirmed by 2% agarose gel electrophoresis.

### Absolute qPCR assay.

Single-band PCR products were purified using the EasyPure PCR purification kit and then inserted into the T vector using the pEASY-T1 simple cloning kit. The recombinant plasmid DNA was transformed into competent cells. Mixtures were uniformly smeared on Luria broth (LB) agar plates and cultured overnight at 37°C. The positive bacterial clones were selected and used for plasmid extraction according to the manufacturer’s instructions for the AxyPrep plasmid DNA minikit (Axygen; APMNP50). The concentration and quality of recombinant plasmid were measured by spectrophotometry (NanoDrop 2000, ThermoFisher) and visualized through 2% agarose gel electrophoresis, respectively. The recombinant plasmid DNA was stored at −80°C until use.

Based on the concentration of recombinant plasmids and the formula developed by Dhanasekaran et al. (85), copy numbers of the recombinant plasmid DNA were determined. The stock solution was 10-fold serially diluted to achieve different concentrations (from 10^8^ to 10^3^ copies/μl) to generate a standard curve.

Absolute quantitative PCR was performed in parallel with samples and corresponding serially diluted standards. The reaction mixture and thermocycler conditions of the PCR were the same as described above. Template DNA was diluted 10-fold before use. Each sample was run in triplicate. The actual copy numbers of specific bacterial 16S rRNA genes in samples were calculated by the threshold cycle (*C_T_*) value relative to the relevant standard curve (86). Each standard curve was constructed by a liner regression of the logarithmic values of the estimated copy number of diluted standards (*x* axis) against the corresponding *C_T_* values (*y* axis). The amplification efficiency (*E*) was related to the slope according to the formula *E* = 10^(−1/slope)^ − 1 (87). The analyses of genus-specific bacterial 16S rRNA gene copy numbers in different samples were performed in SPSS (version 17). Values were normalized with log transformation. The significant differences in the copy numbers of bacteria at different time points were determined by one-way ANOVAs and least significant difference tests on the log-transformed values.

### Data availability.

The raw sequence data reported in this paper have been deposited in the Genome Sequence Archive (88) in the BIG Data Center (89), Beijing Institute of Genomics (BIG), Chinese Academy of Sciences, under accession number CRA001462 and are publicly accessible at http://bigd.big.ac.cn/gsa.

## References

[1] Douglas AE 2015 Multiorganismal insects: diversity and function of resident microorganisms. Annu Rev Entomol 60:17–34. doi:10.1146/annurev-ento-010814-020822. 25341109 PMC4465791

[2] Onchuru TO, Martinez AJ, Ingham CS, Kaltenpoth M 2018 Transmission of mutualistic bacteria in social and gregarious insects. Curr Opin Insect Sci 28:50–58. doi:10.1016/j.cois.2018.05.002. 30551767

[3] Baumann P, Moran NA, Baumann L 1997 The evolution and genetics of aphid endosymbionts. Bioscience 47:12–20. doi:10.2307/1313002.

[4] Hansen AK, Moran NA 2014 The impact of microbial symbionts on host plant utilization by herbivorous insects. Mol Ecol 23:1473–1496. doi:10.1111/mec.12421. 23952067

[5] Shin SC, Kim SH, You H, Kim B, Kim AC, Lee KA, Yoon JH, Ryu JH, Lee WJ 2011 *Drosophila* microbiome modulates host developmental and metabolic homeostasis via insulin signaling. Science 334:670–674. doi:10.1126/science.1212782. 22053049

[6] Lee JB, Park KE, Lee SA, Jang SH, Eo HJ, Jang HA, Kim CH, Ohbayashi T, Matsuura Y, Kikuchi Y, Futahashi R, Fukatsu T, Lee BL 2017 Gut symbiotic bacteria stimulate insect growth and egg production by modulating *hexamerin* and *vitellogenin* gene expression. Dev Comp Immunol 69:12–22. doi:10.1016/j.dci.2016.11.019. 27932027

[7] Wong A-N, Dobson AJ, Douglas AE 2014 Gut microbiota dictates the metabolic response of *Drosophila* to diet. J Exp Biol 217:1894–1901. doi:10.1242/jeb.101725. 24577449 PMC4037322

[8] Wada-Katsumata A, Zurek L, Nalyanya G, Roelofs WL, Zhang AJ, Schal C 2015 Gut bacteria mediate aggregation in the German cockroach. Proc Natl Acad Sci U S A 112:15678–15683. doi:10.1073/pnas.1504031112. 26644557 PMC4697420

[9] Dillon R, Vennard C, Charnley A 2002 A note: gut bacteria produce components of a locus cohesion pheromone. J Appl Microbiol 92:759–763. doi:10.1046/j.1365-2672.2002.01581.x. 11966918

[10] Sharon G, Segal D, Ringo JM, Hefetz A, Zilber-Rosenberg L, Rosenberg E 2010 Commensal bacteria play a role in mating preference of *Drosophila melanogaster*. Proc Natl Acad Sci U S A 107:20051–20056. doi:10.1073/pnas.1009906107. 21041648 PMC2993361

[11] Lize A, McKay R, Lewis Z 2014 Kin recognition in *Drosophila*: the importance of ecology and gut microbiota. ISME J 8:469–477. doi:10.1038/ismej.2013.157. 24030598 PMC3906818

[12] Walsh BS, Heys C, Lewis Z 2017 Gut microbiota influences female choice and fecundity in the nuptial gift-giving species, *Drosophila subobscura* (Diptera: Drosophilidae). Eur J Entomol 114:439–445. doi:10.14411/eje.2017.056.

[13] Berlanga M, Paster BJ, Guerrero R 2009 The taxophysiological paradox: changes in the intestinal microbiota of the xylophagous cockroach *Cryptocercus punctulatus* depending on the physiological state of the host. Int Microbiol 12:227–236. doi:10.2436/20.1501.01.102. 20112227

[14] Chen B, Teh B-S, Sun C, Hu S, Lu X, Boland W, Shao Y 2016 Biodiversity and activity of the gut microbiota across the life history of the insect herbivore *Spodoptera littoralis*. Sci Rep 6:29505. doi:10.1038/srep29505. 27389097 PMC4937375

[15] Amsalem E, Galbraith DA, Cnaani J, Teal PA, Grozinger CM 2015 Conservation and modification of genetic and physiological toolkits underpinning diapause in bumble bee queens. Mol Ecol 24:5596–5615. doi:10.1111/mec.13410. 26453894

[16] Amsalem E, Grozinger CM, Padilla M, Hefetz A 2015 The physiological and genomic bases of bumble bee social behaviour. Adv Insect Physiol 18:37–93. doi:10.1016/bs.aiip.2015.01.001.

[17] Moran NA 2015 Genomics of the honey bee microbiome. Curr Opin Insect Sci 10:22–28. doi:10.1016/j.cois.2015.04.003. 26140264 PMC4484875

[18] Kwong WK, Moran NA 2016 Gut microbial communities of social bees. Nat Rev Microbiol 14:374–384. doi:10.1038/nrmicro.2016.43. 27140688 PMC5648345

[19] Koch H, Schmid-Hempel P 2011 Bacterial communities in central European bumblebees: low diversity and high specificity. Microb Ecol 62:121–133. doi:10.1007/s00248-011-9854-3. 21556885

[20] Martinson VG, Danforth BN, Minckley RL, Rueppell O, Tingek S, Moran NA 2011 A simple and distinctive microbiota associated with honey bees and bumble bees. Mol Ecol 20:619–628. doi:10.1111/j.1365-294X.2010.04959.x. 21175905

[21] Kwong WK, Engel P, Koch H, Moran NA 2014 Genomics and host specialization of honey bee and bumble bee gut symbionts. Proc Natl Acad Sci U S A 111:11509–11514. doi:10.1073/pnas.1405838111. 25053814 PMC4128107

[22] Meeus I, Parmentier L, Billiet A, Maebe K, Nieuwerburgh FV, Deforce D, Wäckers F, Vandamme P, Smagghe G 2015 16S rRNA amplicon sequencing demonstrates that indoor-reared bumblebees (*Bombus terrestris*) harbor a core subset of bacteria normally associated with the wild host. PLoS One 10:e0125152. doi:10.1371/journal.pone.0125152. 25923917 PMC4414509

[23] Kwong WK, Mancenido AL, Moran NA 2017 Immune system stimulation by the native gut microbiota of honey bees. R Soc Open Sci 4:170003. doi:10.1098/rsos.170003. 28386455 PMC5367273

[24] Emery O, Schmidt K, Engel P 2017 Immune system stimulation by the gut symbiont *Frischella perrara* in the honey bee (*Apis mellifera*). Mol Ecol 26:2576–2590. doi:10.1111/mec.14058. 28207182

[25] Engel P, Martinson VG, Moran NA 2012 Functional diversity within the simple gut microbiota of the honey bee. Proc Natl Acad Sci U S A 109:11002–11007. doi:10.1073/pnas.1202970109. 22711827 PMC3390884

[26] Koch H, Schmid-Hempel P 2011 Socially transmitted gut microbiota protect bumble bees against an intestinal parasite. Proc Natl Acad Sci U S A 108:19288–19292. doi:10.1073/pnas.1110474108. 22084077 PMC3228419

[27] Forsgren E, Olofsson TC, Vásquez A, Fries I 2010 Novel lactic acid bacteria inhibiting *Paenibacillus larvae* in honey bee larvae. Apidologie 41:99–108. doi:10.1051/apido/2009065.

[28] Parmentier A, Meeus I, Nieuwerburgh FV, Deforce D, Vandamme P, Smagghe G 2018 A different gut microbial community between larvae and adults of a wild bumblebee nest (*Bombus pascuorum*). Insect Sci 25:66–74. doi:10.1111/1744-7917.12381. 27531583

[29] Tarpy DR, Mattila HR, Newton I 2015 Development of the honey bee gut microbiome throughout the queen-rearing process. Appl Environ Microbiol 81:3182–3191. doi:10.1128/AEM.00307-15. 25724964 PMC4393441

[30] Bosmans L, Pozo MI, Verreth C, Crauwels S, Wäckers F, Jacquemyn H, Lievens B 2018 Hibernation leads to altered gut communities in bumblebee queens (*Bombus terrestris*). Insects 9:E188. doi:10.3390/insects9040188. 30544592 PMC6316087

[31] Powell JE, Eiri D, Moran NA, Rangel J 2018 Modulation of the honey bee queen microbiota: effects of early social contact. PLoS One 13:e0200527. doi:10.1371/journal.pone.0200527. 30001407 PMC6042773

[32] Koch H, Abrol DP, Li JL, Schmid-Hempel P 2013 Diversity and evolutionary patterns of bacterial gut associates of corbiculate bees. Mol Ecol 22:2028–2044. doi:10.1111/mec.12209. 23347062

[33] Tomono T, Sota T 1997 The life and pollination ecology of bumblebees in the alpine zone of central Japan. Jpn J Entomol 65:237–255.

[34] Cameron SA, Lozier JD, Strange JP, Koch JB, Cordes N, Solter LF, Griswold TL 2011 Patterns of widespread decline in North American bumble bees. Proc Natl Acad Sci U S A 108:662–667. doi:10.1073/pnas.1014743108. 21199943 PMC3021065

[35] Goulson D, Nicholls E, Botías C, Rotheray EL 2015 Bee declines driven by combined stress from parasites, pesticides, and lack of flowers. Science 347:1255957. doi:10.1126/science.1255957. 25721506

[36] Naeem M, Liu MJ, Huang JX, Ding GL, Potapov G, Jung CL, An JD 2019 Vulnerability of East Asian bumblebee species to future climate and land cover changes. Agric Ecosyst Environ 277:11–20. doi:10.1016/j.agee.2019.03.002.

[37] Velthuis HHW, Van Doorn A 2006 A century of advances in bumblebee domestication and the economic and environmental aspects of its commercialization for pollination. Apidologie 37:421–451. doi:10.1051/apido:2006019.

[38] Alford DV 1969 A study of the hibernation of bumble bees (Hymenoptera: Bombidae) in southern England. J Anim Ecol 38:149–170. doi:10.2307/2743.

[39] Schmid-Hempel R, Schmid-Hempel P 2000 Female mating frequencies in Bombus spp. from central Europe. Insect Soc 47:36–41. doi:10.1007/s000400050006.

[40] Michener CD 1974 The social behavior of the bees: a comparative study. The Belknap Press of Harvard University Press, Cambridge MA.

[41] Beekman M, Van Stratum P 2000 Does the diapause experience of bumblebee queens *Bombus terrestris* affect colony characteristics? Ecol Entomol 25:1–6. doi:10.1046/j.1365-2311.2000.00235.x.

[42] Evans E, Burns I, Spivak M 2007 Befriending bumble bees: a practical guide to raising local bumble bees. University of Minnesota Extension, Saint Paul, MN.

[43] Bäckhed F, Fraser CM, Ringel Y, Sanders ME, Sartor RB, Sherman PM, Versalovic J, Young V, Finlay BB 2012 Defining a healthy human gut microbiome: current concepts, future directions, and clinical applications. Cell Host Microbe 12:611–622. doi:10.1016/j.chom.2012.10.012. 23159051

[44] Li JL, Powell JE, Guo J, Evans JD, Wu J, Williams P, Lin Q, Moran NA, Zhang ZG 2015 Two gut community enterotypes recur in diverse bumblebee species. Curr Biol 25:R652–R653. doi:10.1016/j.cub.2015.06.031. 26241138

[45] Powell JE, Martinson VG, Urban-Mead K, Moran NA 2014 Routes of acquisition of the gut microbiota of the honey bee *Apis mellifera*. Appl Environ Microbiol 80:7378–7387. doi:10.1128/AEM.01861-14. 25239900 PMC4249178

[46] Nuriel-Ohayon M, Neuman H, Ziv O, Belogolovski A, Barsheshet Y, Bloch N, Uzan A, Lahav R, Peretz A, Frishman S, Hod M, Hadar E, Louzoun Y, Avni O, Koren O 2019 Progesterone increases *Bifidobacterium* relative abundance during late pregnancy. Cell Rep 27:730–736. doi:10.1016/j.celrep.2019.03.075. 30995472

[47] Dahl C, Stanislawski M, Iszatt N, Mandal S, Lozupone C, Clemente JC, Knight R, Stigum H, Eggesbø M 2017 Gut microbiome of mothers delivering prematurely shows reduced diversity and lower relative abundance of *Bifidobacterium* and *Streptococcus*. PLoS One 12:e0184336. doi:10.1371/journal.pone.0184336. 29069100 PMC5656300

[48] Weger BD, Gobet C, Yeung J, Martin E, Jimenez S, Betrisey B, Foata F, Berger B, Balvay A, Foussier A, Charpagne A, Boizet-Bonhoure B, Chou CJ, Naef F, Gachon F 2019 The mouse microbiome is required for sex-specific diurnal rhythms of gene expression and metabolism. Cell Metab 29:362–382. doi:10.1016/j.cmet.2018.09.023. 30344015 PMC6370974

[49] Heys C, Lize A, Colinet H, Price TAR, Prescott M, Ingleby F, Lewis Z 2018 Evidence that the microbiota counteracts male out breeding strategy by inhibiting sexual signaling in females. Front Ecol Evol 6:29. doi:10.3389/fevo.2018.00029.

[50] Wong CAN, Wang QP, Morimoto J, Senior AM, Lihoreau M, Neely GG, Simpson SJ, Ponton F 2017 Gut microbiota modifies olfactory-guided microbial preferences and foraging decisions in *Drosophila*. Curr Biol 27:2397–2404. doi:10.1016/j.cub.2017.07.022. 28756953

[51] Schretter CE, Vielmetter J, Bartos I, Marka Z, Marka S, Argade S, Mazmanian SK 2018 A gut microbial factor modulates locomotor behaviour in *Drosophila*. Nature 563:402–406. doi:10.1038/s41586-018-0634-9. 30356215 PMC6237646

[52] Krieger GM, Duchateau MJ, Van Doorn A, Ibarra F, Francke W, Ayasse M 2006 Identification of queen sex pheromone components of the bumblebee *Bombus terrestris*. J Chem Ecol 32:453–471. doi:10.1007/s10886-005-9013-8. 16555129

[53] Rosengaus RB, Zecher CN, Schultheis KF, Brucker RM, Bordenstein SR 2011 Disruption of the termite gut microbiota and its prolonged consequences for fitness. Appl Environ Microbiol 77:4303–4312. doi:10.1128/AEM.01886-10. 21571887 PMC3127728

[54] Sison-Mangus MP, Mushegian AA, Ebert D 2015 Water fleas require microbiota for survival, growth and reproduction. ISME J 9:59–67. doi:10.1038/ismej.2014.116. 25026374 PMC4274426

[55] Koren O, Goodrich JK, Cullender TC, Spor A, Laitinen K, Bäckhed HK, Gonzalez A, Werner JJ, Angenent LT, Knight R, Bäckhed F, Isolauri E, Salminen S, Ley RE 2012 Host remodeling of the gut microbiome and metabolic changes during pregnancy. Cell 150:470–480. doi:10.1016/j.cell.2012.07.008. 22863002 PMC3505857

[56] Santacruz A, Collado MC, García-Valdés L, Segura MT, Martín-Lagos JA, Anjos T, Martí-Romero M, Lopez RM, Florido J, Campoy C, Sanz Y 2010 Gut microbiota composition is associated with body weight, weight gain and biochemical parameters in pregnant women. Br J Nutr 104:83–92. doi:10.1017/S0007114510000176. 20205964

[57] DiGiulio DB, Callahan BJ, McMurdie PJ, Costello EK, Lyell DJ, Robaczewska A, Sun CL, Goltsman DSA, Wong RJ, Shaw G, Stevenson DK, Holmes SP, Relman DA 2015 Temporal and spatial variation of the human microbiota during pregnancy. Proc Natl Acad Sci U S A 112:11060–11065. doi:10.1073/pnas.1502875112. 26283357 PMC4568272

[58] Billiet A, Meeus I, Van Nieuwerburgh F, Deforce D, Wäckers F, Smagghe G 2016 Impact of sugar syrup and pollen diet on the bacterial diversity in the gut of indoor-reared bumblebees (*Bombus terrestris*). Apidologie 47:548–560. doi:10.1007/s13592-015-0399-1.

[59] Sabaté DC, Carrillo L, Audisio MC 2009 Inhibition of *Paenibacillus larvae* and *Ascosphaera apis* by *Bacillus subtilis* isolated from honeybee gut and honey samples. Res Microbiol 160:193–199. doi:10.1016/j.resmic.2009.03.002. 19358885

[60] Wang M, Zhao WZ, Xu H, Wang ZW, He SY 2015 *Bacillus* in the guts of honey bees (*Apis mellifera*; Hymenoptera: Apidae) mediate changes in amylase values. Eur J Entomol 112:619–624. doi:10.14411/eje.2015.095.

[61] Harzevili ARS, Duffel HV, Dhert P, Swings J, Sorgeloos P 1998 Use of a potential probiotic *Lactococcus lactis* AR21 strain for the enhancement of growth in the rotifer *Brachionus plicatilis* (Muller). Aquac Res 29:411–417. doi:10.1046/j.1365-2109.1998.00217.x.

[62] Beck BR, Kim D, Jeon J, Lee S-M, Kim HK, Kim O-J, Lee JI, Suh BS, Do HK, Lee KH, Holzapfel WH, Hwang JY, Kwon MG, Song SK 2015 The effects of combined dietary probiotics *Lactococcus lactis* BFE920 and *Lactobacillus plantarum* FGL0001 on innate immunity and disease resistance in olive flounder (*Paralichthys olivaceus*). Fish Shellfish Immunol 42:177–183. doi:10.1016/j.fsi.2014.10.035. 25449382

[63] Ceja-Navarro JA, Vega FE, Karaoz U, Zhao H, Jenkins S, Lim HC, Kosina P, Infante F, Northen TR, Brodie EL 2015 Gut microbiota mediate caffeine detoxification in the primary insect pest of coffee. Nat Commun 6:7618. doi:10.1038/ncomms8618. 26173063 PMC4510693

[64] Briones-Roblero CI, Rodríguez-Díaz R, Santiago-Cruz JA, Zúñiga G, Rivera-Orduña FN 2017 Degradation capacities of bacteria and yeasts isolated from the gut of *Dendroctonus rhizophagus* (Curculionidae: Scolytinae). Folia Microbiol (Praha) 62:1–9. doi:10.1007/s12223-016-0469-4. 27544667

[65] Briones-Roblero CI, Hernández-García JA, Gonzalez-Escobedo R, Soto-Robles LV, Rivera-Orduña FN, Zúñiga G 2017 Structure and dynamics of the gut bacterial microbiota of the bark beetle, *Dendroctonus rhizophagus* (Curculionidae: Scolytinae) across their life stages. PLoS One 12:e0175470. doi:10.1371/journal.pone.0175470. 28406998 PMC5391025

[66] Sonoyama K, Fujiwara R, Takemura N, Ogasawara T, Watanabe J, Ito H, Morita T 2009 Response of gut microbiota to fasting and hibernation in Syrian hamsters. Appl Environ Microbiol 75:6451–6456. doi:10.1128/AEM.00692-09. 19700553 PMC2765128

[67] Carey HV, Walters WA, Knight R 2013 Seasonal restructuring of the ground squirrel gut microbiota over the annual hibernation cycle. Am J Physiol Regul Integr Comp Physiol 304:R33–R42. doi:10.1152/ajpregu.00387.2012. 23152108 PMC3543654

[68] Sommer F, Ståhlman M, Ilkayeva O, Arnemo JM, Kindberg J, Josefsson J, Newgard CB, Fröbert O, Bäckhed F 2016 The gut microbiota modulates energy metabolism in the hibernating brown bear *Ursus arctos*. Cell Rep 14:1655–1661. doi:10.1016/j.celrep.2016.01.026. 26854221

[69] Votavová A, Tomčala A, Kofroňová E, Kudzejová M, Šobotník J, Jiroš P, Komzáková O, Valterová I 2015 Seasonal dynamics in the chemistry and structure of the fat bodies of bumblebee queens. PLoS One 10:e0142261. doi:10.1371/journal.pone.0142261. 26559946 PMC4641598

[70] Williams PH, Cameron SA, Hines HM, Cederberg B, Rasmont P 2008 A simplified subgeneric classification of the bumblebees (genus *Bombus*). Apidologie 39:46–74. doi:10.1051/apido:2007052.

[71] Williams PH, Brown MJF, Carolan JC, An JD, Goulson D, Aytkin AM, Best LR, Byvaltsev AM, Cederberg B, Dawson R, Huang JX, Ito M, Monfared A, Raina RH, Schmid-Hempel P, Sheffield CS, Sima P, Xie ZH 2012 Unveiling cryptic species of the bumblebee subgenus *Bombus s. str*. worldwide with COI barcodes (Hymenoptera: Apidae). Syst Biodivers 10:21–56. doi:10.1080/14772000.2012.664574.

[72] Roggenbuck M, Bærholm Schnell I, Blom N, Bælum J, Bertelsen MF, Sicheritz-Pontén T, Sørensen SJ, Gilbert MTP, Graves GR, Hansen LH 2014 The microbiome of new world vultures. Nat Commun 5:5498. doi:10.1038/ncomms6498. 25423494

[73] Caporaso JG, Kuczynski J, Stombaugh J, Bittinger K, Bushman FD, Costello EK, Fierer N, Peña AG, Goodrich JK, Gordon JI, Huttley GA, Kelley ST, Knights D, Koenig JE, Ley RE, Lozupone CA, McDonald D, Muegge BD, Pirrung M, Reeder J, Sevinsky JR, Turnbaugh PJ, Walters WA, Widmann J, Yatsunenko T, Zaneveld J, Knight R 2010 QIIME allows analysis of high-throughput community sequencing data. Nat Methods 7:335–336. doi:10.1038/nmeth.f.303. 20383131 PMC3156573

[74] Edgar R 2013 UPARSE: highly accurate OTU sequences from microbial amplicon reads. Nat Methods 10:996–998. doi:10.1038/nmeth.2604. 23955772

[75] Quast C, Pruesse E, Yilmaz P, Gerken J, Schweer T, Yarza P, Peplies J, Glöckner FO 2013 The SILVA ribosomal RNA gene database project: improved data processing and web-based tools. Nucleic Acids Res 41:D590–D596. doi:10.1093/nar/gks1219. 23193283 PMC3531112

[76] Cole J, Wang Q, Cardenas E, Fish J, Chai B, Farris RJ, Kulam-Syed-Mohideen AS, McGarrell DM, Marsh T, Garrity GM, Tiedje JM 2009 The Ribosomal Database Project: improved alignments and new tools for rRNA analysis. Nucleic Acids Res 37:141–145. doi:10.1093/nar/gkn879. PMC2686447 19004872

[77] Kruskal J 1964 Nonmetric multidimensional scaling: a numerical method. Psychometrika 29:115–129. doi:10.1007/BF02289694.

[78] Ihaka R, Gentleman R 1996 R: a language for data analysis and graphics. J Comput Graph Stat 5:299–314. doi:10.2307/1390807.

[79] Segata N, Izard J, Waldron L, Gevers D, Miropolsky L, Garrett WS, Huttenhower C 2011 Metagenomic biomarker discovery and explanation. Genome Biol 12:R60. doi:10.1186/gb-2011-12-6-r60. 21702898 PMC3218848

[80] Xu LL, Wu J, Guo J, Li JL 2014 Dynamic variation of symbionts in bumblebees during hosts growth and development. Sci Agric Sin 47:2030–2037. doi:10.3864/j.issn.0578-1752.2014.10.017.

[81] Li JL, Qin HR, Wu J, Sadd BM, Wang XH, Evans JD, Peng WJ, Chen YP 2012 The prevalence of parasites and pathogens in Asian honeybees *Apis cerana* in China. PLoS One 7:e47955. doi:10.1371/journal.pone.0047955. 23144838 PMC3492380

[82] Kešnerová L, Mars RAT, Ellegaard KM, Troilo M, Sauer U, Engel P 2017 Disentangling metabolic functions of bacteria in the honey bee gut. PLoS Biol 15:e2003467. doi:10.1371/journal.pbio.2003467. 29232373 PMC5726620

[83] Martinson VG, Moy J, Moran NA 2012 Establishment of characteristic gut bacteria during development of the honey bee worker. Appl Environ Microbiol 78:2830–2840. doi:10.1128/AEM.07810-11. 22307297 PMC3318792

[84] Cariveau DP, Powell EJ, Koch H, Winfree R, Moran NA 2014 Variation in gut microbial communities and its association with pathogen infection in wild bumble bees (*Bombus*). ISME J 8:2369–2379. doi:10.1038/ismej.2014.68. 24763369 PMC4260702

[85] Dhanasekaran S, Doherty TM, Kenneth J 2010 Comparison of different standards for real-time PCR-based absolute quantification. J Immunol Methods 354:34–39. doi:10.1016/j.jim.2010.01.004. 20109462

[86] Yu Y, Lee C, Kim J, Hwang S 2005 Group-specific primer and probe sets to detect methanogenic communities using quantitative real-time polymerase chain reaction. Biotechnol Bioeng 89:670–679. doi:10.1002/bit.20347. 15696537

[87] Lee C, Kim J, Shin SG, Hwang S 2006 Absolute and relative QPCR quantification of plasmid copy number in *Escherichia coli*. J Biotechnol 123:273–280. doi:10.1016/j.jbiotec.2005.11.014. 16388869

[88] Wang Y, Song F, Zhu J, Zhang S, Yang Y, Chen T, Tang B, Dong L, Ding N, Zhang Q, Bai Z, Dong X, Chen H, Sun M, Zhai S, Sun Y, Yu L, Lan L, Xiao J, Fang X, Lei H, Zhang Z, Zhao W 2017 GSA: genome sequence archive. Genomics Proteomics Bioinformatics 15:14–18. doi:10.1016/j.gpb.2017.01.001. 28387199 PMC5339404

[89] BIG Data Center Members. 2019 Database resources of the BIG Data Center in 2019. Nucleic Acids Res 47 (D1):D8–D14. doi:10.1093/nar/gky993. 30365034 PMC6323991

