# Supplementary figures and images for: Dynamic Changes of Gut Microbial Communities of Bumble Bee Queens through Important Life Stages
Source: mSystems. 2019 Dec 10;4(6):e00631-19. doi: 10.1128/mSystems.00631-19 (PMC6906740; doi:10.1128/mSystems.00631-19)

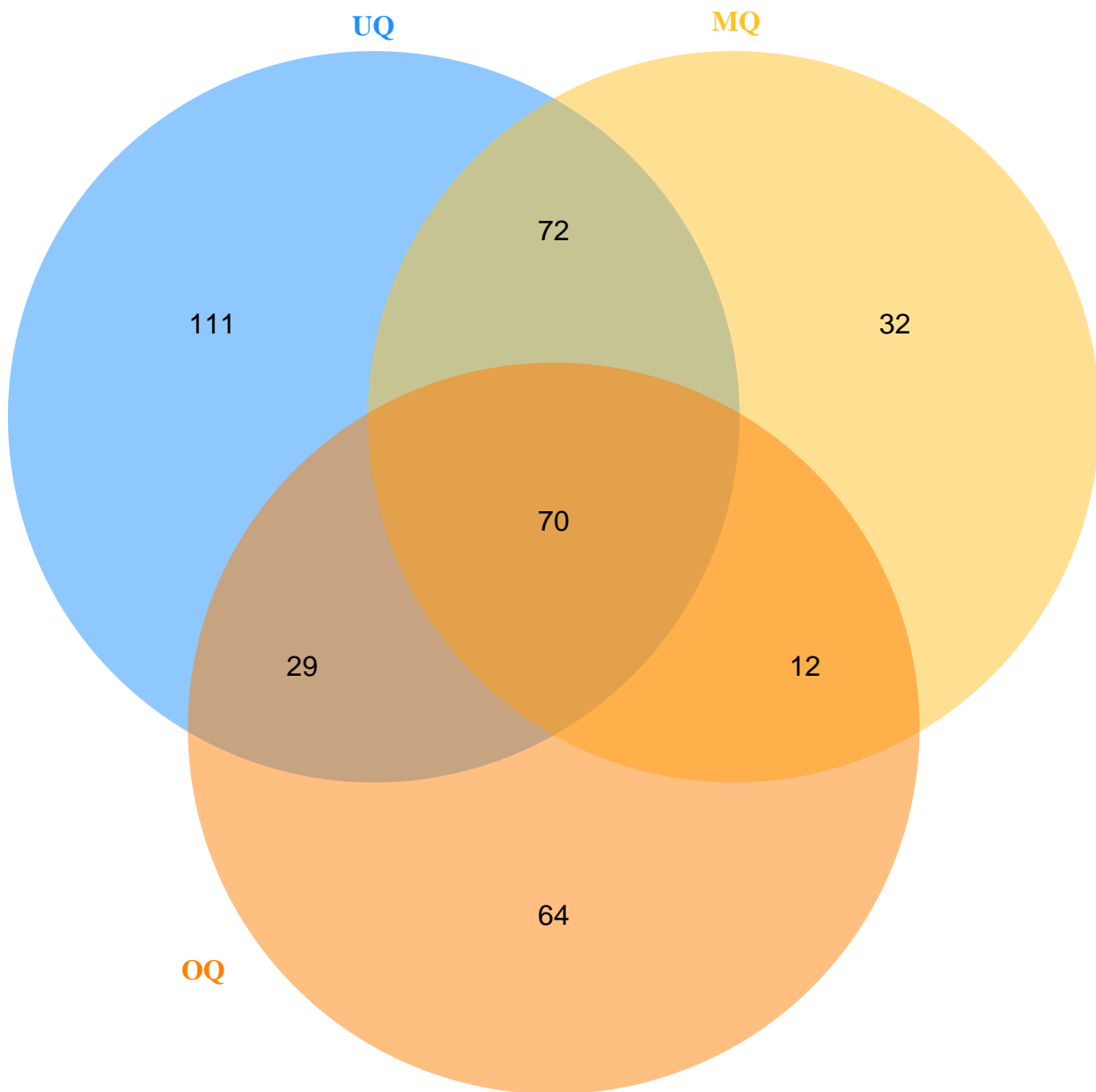

Supplement: FIG S1 [file mSystems.00631-19-sf001.pdf]

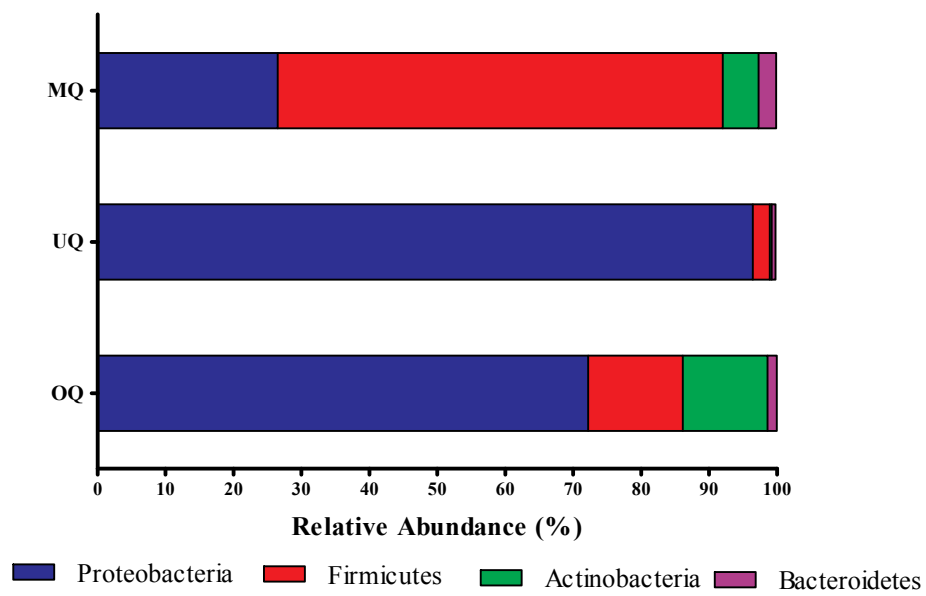

Supplement: FIG S2 [file mSystems.00631-19-sf002.pdf]

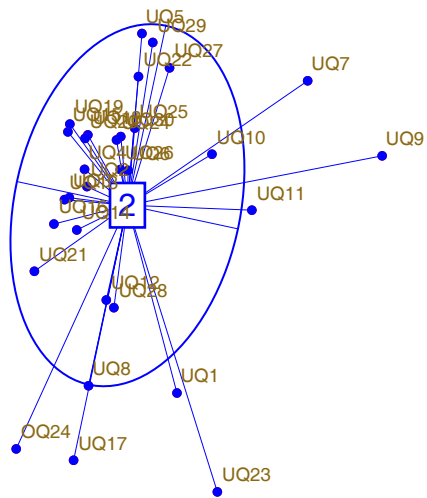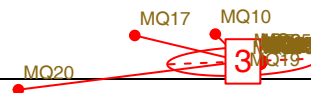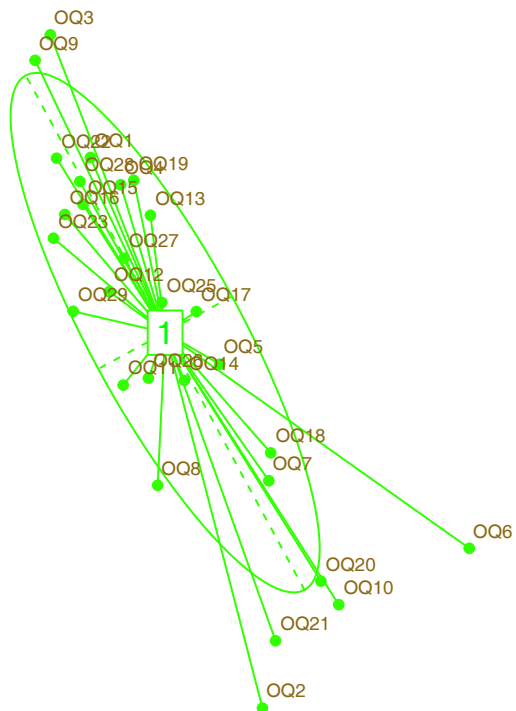

Supplement: FIG S3 [file mSystems.00631-19-sf003.pdf]
